# Evolution of microRNA (miRNA) Structure and Function in Plants and Animals: Relevance to Aging and Disease

**DOI:** 10.4172/2329-8847.1000119

**Published:** 2014-04-11

**Authors:** Aileen I Pogue, Christian Clement, James M Hill, Walter J Lukiw

**Affiliations:** 1 Alchem Biotek, Toronto ON, M5S 1A8, Canada; 2 Departments of Ophthalmology, LSU Neuroscience Center, USA; 3 Departments of Microbiology, LSU Neuroscience Center, USA; 4 Departments of Pharmacology, LSU Neuroscience Center, USA; 5 Departments of Neurology, LSU Neuroscience Center, USA

## Introduction

Micro RNAs (miRNAs) constitute a family of small, single-stranded RNAs (ssRNAs) involved in the post-transcriptional regulation of gene expression and in ultimately shaping the transcriptome of a cell. While our views on the biological significance and relevance of miRNA signaling continue to evolve, it is now generally recognized that the principal action of miRNA in all species of plants and animals is to recognize, and then bind, via highly selective hydrogen bonding, to specific complementary ribonucleotide targets in the 3’ prime untranslated region (3’-UTR) of specific messenger RNAs (mRNAs), and in doing so, down-regulate their expression [1–10]. Although miRNAs are considered to be critically important epigenetic regulators of gene expression in eukaryotic development, aging and disease, it is not often appreciated that these ssRNAs: (i) operate ubiquitously and sometimes interactively throughout the plant and animal kingdoms; (ii) are highly selected in their ribonucleotide sequence; (iii) are highly selected in their cell and tissue specificity, (iv) represent an epigenetic signaling system that is evolutionarily ancient; (v) are the smallest yet characterized ribonucleic acid carriers of highly selective genetic regulatory information; (vi) possess highly similar structural and functional features when compared to minimalist plant pathogens known as viroids; (vii) are the most abundant nucleic acids contained in human extracellular fluid (ECF) and cerebrospinal fluid (CSF); and (viii) as major components of the ECF, CSF and blood serum may spread both homeostatic and pathological signaling among neighboring cells and tissues, and perhaps even between individual organisms or species [11–13]. This communication will briefly review some of the more overlooked aspects of the structure, function and mechanism of these fascinating small non-coding RNAs (sncRNAs) in plants and animals, with special emphasis on human central nervous system (CNS) disease, and with specific relevance to Alzheimer disease (AD) wherever possible.

## The Unique Sequence Structure, Selectivity, Tissue Specificity and Stability of miRNAs

Elementary ribonucleic acid sequence analysis and bioinformatics predict that a ‘typical’ 22 nucleotide ssRNA that is comprised of 4 different ribonucleotides (adenine, guanine, cytosine and uridine; A,G,C,U) could have over 10^13^ possible sequence combinations. The experimental observation that there are typically only about 2×10^3^ different miRNAs so far identified in all eukaryotic tissues examined, and that miRNAs are highly developmental stage-, tissue- and cell-specific, even in adjacent cell types suggests an extremely high evolutionary selection pressure to use only specific miRNA sequences that will yield biologically productive miRNA-mRNA interactions [1– 4,14–16]. In fact, extensive studies using miRNA array-, Northern dot blot-, RNA-sequencing, RT-PCR and bioinformatics-based analyses on small ssRNAs suggest that human brain cells probably utilize less than 10^2^ abundant species of miRNA [17–20] and that only a relatively small fraction of these are misregulated in AD and other neurodegenerative disorders with an inflammatory component [17,19,21,22; unpublished observations].

## Abundance and Stability of miRNAs

**Figure 1:**
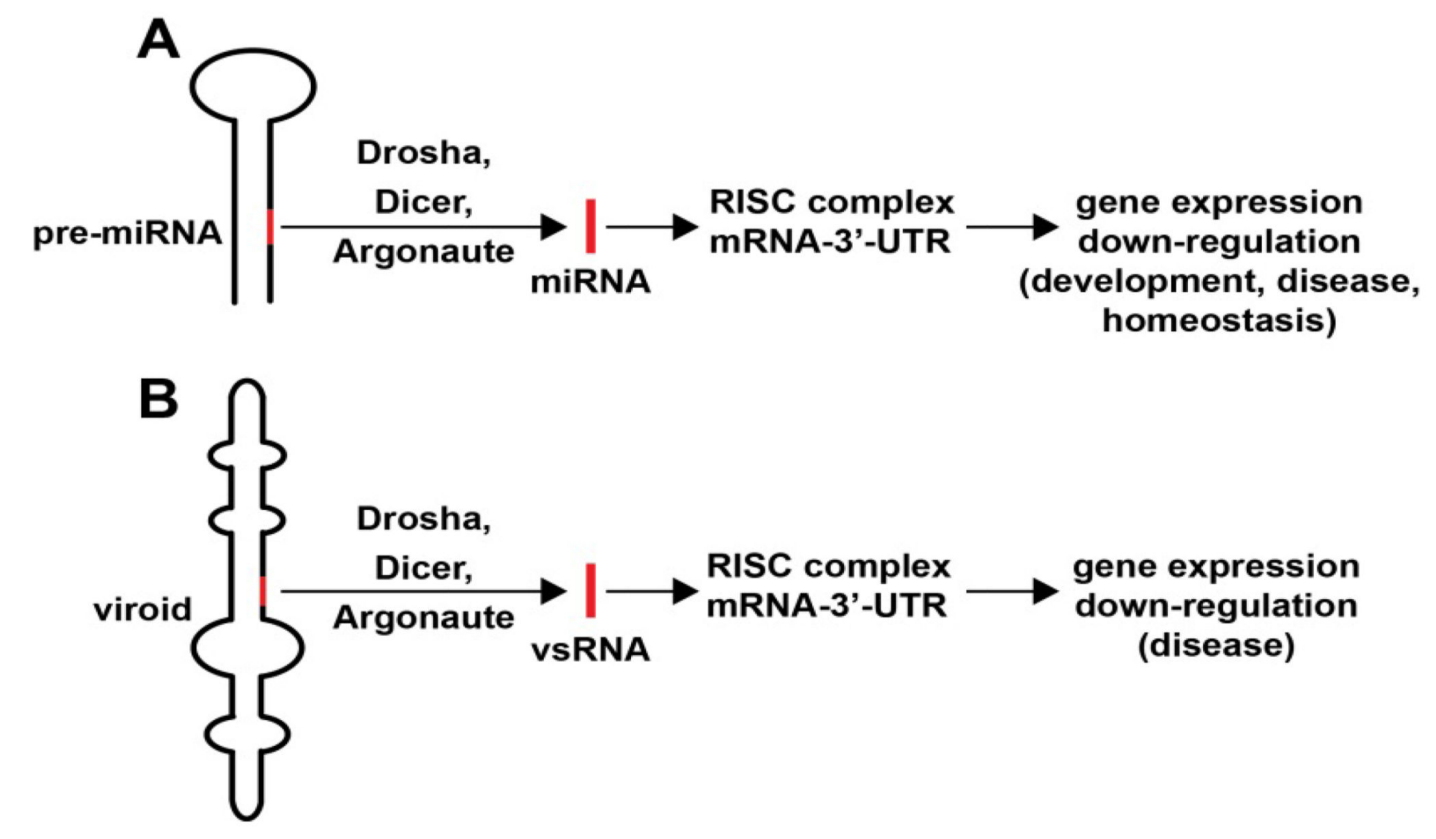
Similarities in miRNA and viroid structure and function This highly schematicized figure underscores the remarkable similarities between the structure and function of miRNA and viroids; **(A)** a typical 75–110 nucleotide primary micro RNA (pri-miRNA) ‘hairpin’ containing an endogenous 21–25 nt miRNA that yields a mature miRNA (red bar) after Drosha- and Dicer-mediated excision and processing; the mature miRNA next associates with a cytoplasmic RNA-induced silencing complex (RISC) and a target mRNA-3’-UTR to degrade and down-regulate expression of that target mRNA, with subsequent effects on the expression of genes involved in homeostasis, development and disease; **(B)** analogously, a ~246–401 nucleotide closed circular viroid, also containing extensive intra-strand base pairings, dsRNA and stem-loop structures, typically contains a 21–25 nucleotide viroid-specific RNA (vsRNA; red bar) that after host Drosha/Dicer-based processing yields a mature vsRNA; as is the case for miRNAs this vsRNA subsequently targets the RISC and mRNA-3’-UTR complex, down-regulating gene expression to induce progressive developmental and age-related disease in plants [26,27,40,44,72]. In both cases larger miRNA or vsRNA precursors are processed by an RNase III of the family of Dicer-like proteins to generate smaller ‘infectious’ ssRNA species; these sizes are similar to endogenous small interfering RNA (as mature vsRNA or miRNA) to alter the viroid-dependent gene expression in the host plant by viroids, or of miRNA-mRNA processing in animal species including humans [14,26,27,37,42,44,72]. While naked, mature RNAs such as miRNAs and vsRNAs have relatively short half-lives *in vitro* (for example human neuronal miRNAs appear to be highly labile [27,43], stabilities may be greatly extended by single- or double-stranded RNA-binding proteins, by complex secondary structures, by RNA circularization, by containment in protease- and RNase-resistant vesicles, or by combinations of these and other factors [23,24,26,27,43]. Interestingly, viroids, at about one one-thousandth the size of the smallest known ssRNA virus (see text), are the smallest known self-replicating pathogens of all living species, and both the plant and animal kingdoms have adopted similar ssRNA strategies to store and transmit only the most essential genetic regulatory information in the propagation of either pathological or homeostatic signals. The potential for interaction between various vsRNAs and miRNAs in their hosts, if any, among diverse species of the plant and animal kingdom is currently not known.

The abundance, complexity and speciation of highly specific miRNAs may vary among human populations in health and disease, and as such, have high potential for the diagnosis of disease [15,16,21]. Like mRNA, miRNAs appear to adopt the same stability rules involving adenine-uridine (AU) rich elements (AREs) in their linear ribonucleotide sequence, and a higher ARE content in miRNAs is generally associated with shorter miRNA half-life. Conversely the absence of AU or UA dinucleotide elements may confer miRNA stability and lengthen miRNA half-life [16,23–27]. Interestingly, while mammalian brain and retinal miRNAs in particular may have in general a relatively short half-life measured in the range of several hours, miRNA half-lives may be considerably extended by miRNA-binding proteins, the adoption of extensive miRNA secondary and tertiary structures, circularization, or by combinations of these RNAse-evading strategies [11,13,16,26,27]. Within miRNA precursors, virtually all of the miRNA sequence base-pairs with complementary sequences in other parts of the same molecule to form double-stranded RNA (dsRNA) structures that are more resistant to degradation than ssRNA alone [27,28] (Figure 1).

## miRNAs Occur Ubiquitously in Plants and Animals

While different miRNAs seem to be abundant in different cells, tissues and plant and animals species, at times primary structures may be very highly conserved as demonstrated by a presence or absence of certain ribonucleotides at specific positions in the miRNA and in the flanking regions of their precursors. Such miRNA sequences may contain fingerprints for conservation across multiple plant and animal species, and these fingerprints represent some of the most highly conserved nucleic acid sequences known [13,14,28,29]. To cite just one recent example, using novel genome-wide computational approaches to detect miRNAs based on both sequence and structure alignment across the plant and animal kingdoms, the miRNA-854 family has been shown to be expressed in *Arabidopsis thaliana, Caenorhabditis elegans*, *Mus musculus* and *Homo sapiens*. Interestingly, across these diverse species, miRNA-854 commonly targets the uridylate binding protein 1b (*UBP1b*) mRNA 3’-UTR, and UBP1b normally encodes a member of a heterogeneous nuclear RNA binding protein-1 (hnRNP-1) gene family [13,14]. This indicates an evolutionary common origin of miRNA-854 as a regulator of the basal eukaryotic transcription mechanism in both plants and animals for many hundreds of millions of years (*Arabidopsis thaliana* - *Homo sapiens* divergence about 1.5 billion years; [13,14,30,31]. While secondary and tertiary structures may also be conserved among multiple miRNAs, and internal stems, loops and mis-paired RNA ‘bulges’ very commonly appear at specifically conserved positions in many pre-miRNA sequences, the actual number of possible miRNA structural configurations may be somewhat limited by the small linear size (~21–25 nucleotides) of the miRNA itself [14,19,28,32] (Figure 1).

## miRNAs and Viroids have Extensive Similarity in Structure and Function

miRNAs are the smallest yet identified carriers of highly selective genetic regulatory information in plants and animals. Their ribonucleotide sequences further define and regulate the expression of a relatively discrete subset of cellular mRNAs with which they may interact, thus defining a highly complex and interactive gene regulation network [1–6,33–35]. Across their ~21–25 ribonucleotide sequences miRNAs carry encoded genetic signals which may be transmitted via their unique molecular shape, nucleic acid topology and charge density along their lengths. Interestingly, miRNAs are typically less than about one one-thousandth of the size of a ‘typical virus’; the smallest ssRNA viruses known, in terms of genome size are retroviruses (such as Rous Sarcoma virus with a 3.5×10^4^ nucleotide genome; http://www.ictvonline.org/) [36]. Seven orders, 96 families, 22 subfamilies, 420 genera, and about 2,618 species of viruses have been recently classified by the International Committee on taxonomy of viruses (ICTV), including 5 orders and 47 families of RNA viruses, both double and single stranded [36,37]. Curiously, there is approximately the same number of species of viruses as there are all currently known mammalian miRNAs (numbering about ~2600).

## miRNAs and Viroids

Smaller than any known ssRNA viruses are viroids, a family of about 30 plant pathogens consisting of circular ssRNAs ranging in size from 246 to 401 nucleotides. Viroids are of evolutionary, virological and biological interest since they may represent living fossils of pre-cellular evolution in a hypothetical RNA world [13,38–42]. One of the smallest and first discovered viroids is the potato spindle tuber viroid (PSTV), a circular ssRNA which causes infectious disease in potato plants (chiefly *Solanum tuberosum*), and remains an important agricultural and economic concern throughout the world (Figure 1) [13,38–40]. Viroids represent a class of truly minimalist plant pathogens and it remains controversial whether they are a biological oddity, an evolutionary fossil or a highly evolved plant pathogen [39–42]. Viroids are transcribed by a unidirectional nucleic acid rolling-circle mechanism in the host plant’s chloroplast (for the genus *Avsunviroidae*) or nuclei (for the genus *Pospiviroidae*). Relatively recent findings indicate that viroid infection is associated with the appearance of small viroid-specific RNAs (vsRNAs), approximately 21 to 25 nucleotides in size, processed by an RNase III of the family of Dicer-like proteins from a pre-viroid precursor [37] (Figure 1). Abundant in viroid-infected plant species, vsRNAs have sizes similar to naturally occurring endogenous small interfering RNAs and miRNAs, and appear to permanently alter the normal cultivar- and viroid-dependent gene expression patterns in the host [37–42]. Highly analogous to miRNAs, viroid ssRNAs encode no proteins, have no protective protein capsid or coat, do not reverse-transcribe into DNA when they replicate, and are significantly inducible by external stressors, including neurotoxic factors in the environment [18,37–39]. As naked, infectious vsRNA molecules viroid replication requires an endogenous, host-supplied polymerase, initially generated as an internally complementary, double-stranded precursor RNA (previroid) structure from which a mature ssRNA is excised by RNAse III-type Dicer and related riboenzymes (Figure 1). As far as is currently known, miRNAs do not replicate *in vivo,* as they are probably physically too small to do so, and *in vitro* require a linker DNA or RNA to efficiently copy themselves using exogenous polymerase systems and replication cofactors. The circularization of pre-viroids, pri-miRNAs, anti-miRNAs or miRNAs, and the formation of complex higher order secondary and tertiary structures, may stabilize them and protect against rapid RNA degradation in cellular environments normally enriched in RNAse and associated depolymerization systems [11,13,41–44].

## miRNAs are Abundant in Systemic Fluids, Including the ECF and CSF of the Human CNS

Emerging studies on human brain extracellular fluid (ECF; a cell-free preparation of the extracellular fluid that encircles human brain cells) and cerebrospinal fluid (CSF; the fluid that surrounds the brain and spinal cord) indicate that miRNAs are the most abundant nucleic acids contained within the circulating fluids of the human CNS [21,45]. Increases in specific miRNAs, confirmed independently using both miRNA arrays (LC Sciences, Houston TX) and reconfirmed using a highly sensitive LED-Northern dot-blot assay, indicate that several NF-κB-sensitive miRNAs detected in ECF and CSF were formerly identified to be up-regulated in brain anatomical regions targeted by AD [15,21,45]. These miRNAs strongly associate with the progressive spreading of inflammatory neurodegeneration [45–48]. The ECF- and CSF-enriched miRNA species include, prominently, miRNA-9, miRNA-34a, miRNA-125b, miRNA-146a and miRNA-155 and others, and their selective enrichment in circulating CSF and ECF in AD suggests that they may be involved in the modulation or proliferation of miRNA-triggered pathogenic signaling throughout the brain and CNS. If abundance is any indication of importance, then these miRNAs may be performing significant pathogenic signaling and disease-transmission functions via the circulation of these CNS fluids. Recent studies further indicate the importance of these and other miRNAs in the regulation of human brain endothelial and epithelial cell-barrier functions [49,50]. A corollary to this is that over-expressed miRNAs may be involved in disease spreading as they pass so easily out of the cell, into adjacent cells, and throughout biological fluids [21,33,45–47,49–54]. Importantly, despite variability in the analysis of AD CSF from many diverse human population samples, increases in the pro-inflammatory miRNA-146a have been reported by multiple independent AD miRNA researchers, and miRNA-146a has also been detected to be significantly up-regulated in transgenic AD murine models, in rare human and rodent prion diseases, epilepsy, and in other neurological disorders associated with progressive inflammatory neurodegeneration [35,46–48,52,54– 56]. Cumulatively these data support the hypothesis that paracrine or endocrine effects of miRNAs originating in stressed constituent cells of the human neurovascular unit may contribute to “spreading events” of AD pathology, and appear to be a unifying characteristic of progressive neurodegenerative disorders [57,58]. As further discussed below; these data additionally suggest that peripheral anti-miRNA strategies may be therapeutically useful in containing the spread of neuropathology not only in AD but in other progressive inflammatory degenerative diseases [25,59].

Interestingly, microRNAs are also abundant in the systemic circulation in humans and may have potential diagnostic value, especially in the earliest stages of AD and other neurological diseases [59,60]. It is not well understood if, like miRNAs, vsRNAs are abundant in the systemic fluids transported by the xylem ‘*vessel cell system*’ in diseased plants; the high cellulose-, xylan-, and lignin-enriched cell walls located outside of the cell membrane of plant cells may prevent easy access of vsRNA to cytoplasmic compartments [13,38–41].

## miRNA and the Spreading of CNS Disease

Alzheimer’s disease (AD), as the prototypical example of a progressive human neurological disease whose most significant risk factor is aging, appears to originate in the limbic system of the human brain and subsequently spreads radially from the hippocampal CA1 and superior temporal lobe regions into more distal lobes of the brain, including, eventually, the frontal and parietal poles and primary visual cortex [8,25,48,60,61]. Hence, a classical neuropathological feature of AD is this progressive and propagating nature of AD inflammatory neuropathology, including the age-related deposition of Aβ42 peptides as insoluble senile plaque deposits [62–66]. The mechanism of AD spreading throughout these anatomical regions of the human brain involved in cognition and memory are not well understood. The small size of miRNAs, the recent identification of miRNA protective proteins, the circularization of miRNAs into complex secondary and tertiary structures, and the fact that miRNAs may concentrate into small membrane-encased secretory vesicles suggest that miRNAs may represent novel transmissible factors for paracrine, endocrine and related forms of intercellular and inter-tissue communication and potential disease spreading amongst CNS [67–69; unpublished observations]. Interestingly, miRNAs are secreted into the surrounding cell culture growth medium when human primary neuronal-glial cocultures are stressed with cytokines (such as IL-1β and TNFα) and other AD-relevant stressors [70,71]. When control neuronal-glial co-cultures are subsequently treated with this surrounding ‘conditioned growth medium’, these brain cells take on characteristics of the stressed cells from which the surrounding ‘conditioned growth medium’ was derived [71; unpublished observations]. Understanding the mechanism for the potential pathological proliferation of AD by miRNAs may of course expose unique opportunities for the development of novel diagnostic techniques, including anti-miRNA-based therapeutic strategies, not only for AD but also for related neurodegenerative disorders such as Parkinson’s disease, Down’s syndrome (trisomy 21), fronto-temporal dementia, prion disease, and other progressive neurological diseases with an inflammatory component.

## Concluding Remarks

In summary, at least 7 independent lines of research currently support the contention that miRNAs may be involved in the pathological spreading of AD, including the following empirical observations: (i) their similarity in structure, function and pathological mechanism to viroids, the smallest infectious agent known, which spread degenerative disease in plants; (ii) that the growth medium of stressed primary human brain cells contain elevated pro-inflammatory miRNA levels; (iii) that these same potentially pathogenic miRNAs are abundant in AD ECF and CSF; (iv) that the pathogenic miRNAs found to be elevated in AD brain tissues are also abundant in AD ECF and CSF; (v) that miRNA-containing growth medium from stressed primary human brain cells can *in vitro* down-regulate pro-inflammatory regulatory glycoproteins and receptors such as complement factor H (CFH) and the triggering receptor in myeloid cells (TREM2; CFH and TREM2 are known to be down-regulated in AD); (vi) that when individual miRNAs of the same type found to be up-regulated in conditioned growth medium are added to normal human brain cells in primary culture, AD-type changes rapidly ensue; and (vii) that anti-miRNA (AM) strategies can quench the phenomenon of miRNA-mediated inflammatory spreading in stressed primary human brain cells [21,33,52,53,60,62,64,70–73].

What is also noteworthy is that miRNAs may be highly mobile not only between cells and tissues but between species, carrying genetic regulatory information outside of the cells and tissues from which they were initially generated [11,13,21,33,73]. While several studies need to be independently replicated, the very recent observations of (1) plant-derived miRNAs being enriched in humans who consume these plants in their diet; and (2) human microbiome-derived sncRNA and miRNA, and plant miRNA translocation across endothelial barriers, between cells and tissues, and even between individual species, indicates that human neurobiology may be significantly impacted by the actions of microbiome-mediated or externally-derived sncRNA or miRNA trafficking, and the integration of a cell, tissue or even an entire organism into its local environment [11–13,21,50,71,73; unpublished].

## Summary

Our concepts of the structure and function of sncRNA, including miRNA and vsRNA and their regulatory mechanisms in development and aging, and how they fit into the fascinating realm of dysfunctional RNA metabolism in diseases of plants and animals, and more specifically human neurological diseases such as AD, continues to evolve. Similarities in the molecular-genetics of miRNA and viroid generation, structure and function suggest that some very basal and conserved mechanisms of disease processes have been preserved across the evolution of multiple and diverse plant and animal species. Indeed, pathogenically up-regulated miRNAs and sncRNAs can be considered as an ancient, epigenetic system used to down-regulate specific mRNAs and their expression, and this elegant regulatory system has been maintained across a plant and animal species divergence that occurred some 1.5 billion years ago [13,14,30,31,69]. Significantly up-regulated miRNAs in neurodegenerative disorders such as AD may help explain the large number of brain gene mRNAs, essential for homeostatic brain activity, that are observed to be progressively down-regulated in AD susceptible anatomical regions as AD advances [62,70,74–77]. In the case of AD, and perhaps other progressive age-related neurological diseases, miRNA- and/or anti-miRNA-directed therapeutics represent an obvious choice for future pharmacological treatment strategies, and these would enable the diseased CNS to regain more normalized and homeostatic gene expression functions.
